# Multicomponent Exercise and Functional Fitness: Strategies for Fall Prevention in Aging Women

**DOI:** 10.3390/sports13060159

**Published:** 2025-05-24

**Authors:** André Schneider, Luciano Bernardes Leite, José Teixeira, Pedro Forte, Tiago M. Barbosa, António M. Monteiro

**Affiliations:** 1 Department of Sports Sciences, Polytechnic Institute of Bragança, 5300-253 Bragança, Portugal; andrecschneider@gmail.com (A.S.); pedromiguelforte@gmail.com (P.F.); barbosa@ipb.pt (T.M.B.); 2 Department of Physical Education, Federal University of Viçosa, Viçosa 6570-900, Brazil; luciano.leite@ufv.br; 3 Department of Sports Sciences, Polytechnic of Guarda, 6300-559 Guarda, Portugal; zeteixeira1991@gmail.com; 4 Department of Sports Sciences, Polytechnic of Cávado and Ave, 4800-058 Guimarães, Portugal; 5 Research Center for Active Living and Wellbeing (Livewell), Polytechnic Institute of Bragança, 5300-253 Bragança, Portugal; 6 Departament of Sports, ISCE Douro, 4560-000 Penafiel, Portugal

**Keywords:** multicomponent exercise, functional fitness, fall prevention, aging women, strength and balance training

## Abstract

Aging is associated with physiological changes that increase the risk of falls, impacting functional independence and quality of life. Multicomponent exercise training has emerged as an effective strategy for mitigating these risks by enhancing strength, balance, flexibility, and aerobic capacity. This study aimed to evaluate the effects of a 30-week multicomponent training program on functional fitness and fall prevention in older women. A parallel, single-blind randomized controlled trial was conducted with 40 participants (aged ≥ 65 years), divided into an exercise group and a control group. The intervention combined strength, balance, coordination, and aerobic training, following international exercise guidelines for older adults. Functional fitness was assessed using validated tests, including the Timed Up and Go (TUG) test, lower limb strength, flexibility, and aerobic endurance measures. Results demonstrated significant improvements in the intervention group, particularly in TUG performance (*p* < 0.001), lower limb strength (*p* < 0.001), and flexibility (*p* < 0.05), indicating enhanced mobility and reduced fall risk. These findings reinforce the importance of structured, multicomponent training programs for aging populations, particularly women, who experience greater musculoskeletal decline due to menopause-related hormonal changes. Future research should explore long-term retention of benefits and optimize intervention strategies. This study highlights the critical role of tailored exercise programs in promoting active aging, improving functional capacity, and reducing healthcare burdens associated with fall-related injuries.

## 1. Introduction

Population aging is a global reality that has significantly reshaped demographic, social, and economic structures worldwide [1]. According to United Nations projections, by 2050 the number of people aged 60 and over will exceed 2 billion, representing more than 20% of the global population [2]. In Portugal, this trend is even more pronounced, as the country ranks among the oldest populations in Europe. In 2018, approximately 22% of the population was aged 65 or older [3], with projections indicating an increase to 45% by 2050 [4]. This demographic shift presents major public health challenges, particularly concerning the maintenance of quality of life and functional independence among older adults [5].

Aging is accompanied by various physiological changes that negatively affect individuals’ functional capacity and autonomy [6]. Among the most notable are the progressive loss of muscle mass (sarcopenia) [7], decreased bone density (osteoporosis) [8], and declines in sensory and motor functions [9,10], as well as reduced cardiorespiratory fitness [11]. These changes make older adults more susceptible to multiple health issues, including impaired mobility [12], increased incidence of chronic diseases [13], and, most critically, a heightened risk of falls [14,15].

It is important to highlight, however, that many of these impairments—such as loss of muscle mass, reduced balance, and decreased cardiorespiratory fitness—are not solely caused by the aging process itself but are strongly associated with a sedentary lifestyle and reduced physical activity levels [16]. This distinction is critical, as these disuse-related declines are largely modifiable and can be mitigated through regular, structured exercise interventions [17]. Recognizing inactivity as a key contributing factor reinforces the role of physical activity in preserving function and preventing falls in older adults [16,17].

Falls are among the most serious and prevalent health concerns in the aging population. Their consequences go beyond physical injuries—such as fractures and head trauma—to include psychological effects, such as fear of falling, and social consequences, including loss of independence and the need for institutional care [18,19,20,21]. In Portugal, for instance, between 2000 and 2013, three out of every 100 hospitalizations of individuals over 65 years were due to falls [22]. Moreover, falls are a leading cause of hospitalizations and deaths in older adults, imposing a substantial burden on healthcare and social support systems [23]. Although most falls are non-fatal, they are associated with significant morbidity and greater functional decline. Approximately 0.6% of fall-related hospitalizations in Portugal result in death [24]. In light of this impact, fall prevention has become a priority in promoting the health and well-being of aging populations [14,25].

In response to this pressing issue, non-pharmacological strategies—particularly physical activity and exercise—have gained prominence in fall prevention and the promotion of healthy aging. Regular physical activity is widely recognized as one of the most effective interventions for reducing the risks associated with aging [26], especially in decreasing fall incidence in older adults [27,28]. Various forms of exercise, including aerobic activities, resistance training, and balance exercises, offer specific benefits that contribute to the preservation of functional capacity, independence, and fall risk reduction [29,30,31,32]. Aerobic exercise improves cardiorespiratory capacity and endurance [33,34]; resistance training increases muscle strength and helps combat sarcopenia [35,36]; and balance and coordination exercises enhance postural control and responsiveness, thereby reducing fall risk [37,38].

To address the multifactorial nature of fall risk comprehensively, multicomponent exercise programs have emerged as a particularly effective strategy [39,40]. These programs integrate different exercise modalities—such as strength, balance, flexibility, and endurance—into a unified intervention, allowing older adults to work on multiple aspects of fitness simultaneously [41]. Additionally, these programs can be adapted to individual capacities, making them safe and accessible for people with varying physical abilities [42]. Evidence shows that regular participation in multicomponent programs significantly reduces fall risk by enhancing postural control, motor coordination, and responses to external perturbations, which are essential for stability and mobility in later life [43,44,45,46,47,48].

Despite the proven benefits of multicomponent training in fall prevention, there remains a relative lack of research focused specifically on older women. Many clinical trials include mixed-sex samples or do not report sex-specific outcomes, limiting the generalizability of findings to this population. Some studies suggest that hormonal and musculoskeletal changes related to menopause may influence training responses, underscoring the importance of tailored interventions for postmenopausal women [49,50].

In this regard, strength training enhances muscle mass and functional capacity, reducing lower limb weakness, while balance and agility exercises improve proprioception and reactivity to unexpected events, thus preventing falls [41]. Understanding the benefits and implementation of multicomponent training [51] enables health professionals, physical educators, and public health officials to develop effective, evidence-based interventions tailored to the needs of older adults. Such strategies not only promote healthy and active aging, but also contribute to the sustainability of healthcare systems in an aging society [52].

Aging in women is associated with accelerated losses in muscle mass and bone mineral density, primarily due to the decline in estrogen levels during menopause [53]. These changes increase the risk of falls and fractures, especially in areas such as the hip and spine, highlighting the need for targeted preventive approaches [54]. Moreover, the decline in balance, proprioception, and lower limb strength further compromises postural stability [55]. Therefore, interventions involving structured exercise—particularly multicomponent programs—are essential to mitigate these impacts and support safety and autonomy in older women [49,50,56,57].

Given these challenges, it is essential to investigate evidence-based interventions capable of counteracting the physiological impacts of aging in women. The present study, therefore, aims to evaluate the effects of a multicomponent exercise program on fall prevention and functional fitness in older women, with particular attention to the Portuguese population. By addressing key factors such as balance, strength, and mobility—domains commonly impaired with age—this research seeks to contribute to the development of accessible, non-pharmacological strategies that foster autonomy, safety, and quality of life in this vulnerable group.

## 2. Materials and Methods

### 2.1. Study Design

This parallel-group, single-blind randomized controlled trial (RCT), with a total duration of 30 weeks, was approved by the Ethics Committee of the Polytechnic Institute of Bragança (IPB) under protocol number 2067313 and registered at ClinicalTrials.gov (identifier: NCT06843486). The study protocol adhered to the Standard Protocol Items: Recommendations for Interventional Trials (SPIRIT) guidelines [58] and was conducted in accordance with the principles outlined in the Declaration of Helsinki. The reporting followed the Consolidated Standards of Reporting Trials (CONSORT) guidelines [59].

### 2.2. Sample

The study was initiated following ethical approval from the Ethics Committee of the Polytechnic Institute of Bragança (IPB), under protocol number 2067313. Participant recruitment was conducted based on specific inclusion and exclusion criteria. Eligible participants were community-dwelling women aged 65 years or older, without psychological or physical conditions that could impair their participation in the exercise program or assessments. They were also required to have adequate visual and auditory acuity to perform the activities safely and effectively, and not rely on assistive devices for mobility or demonstrate notable difficulties in performing activities of daily living.

Exclusion criteria included: women under the age of 65; those with psychological or physical disorders that would limit their participation in training or testing; critical visual and/or auditory impairments that could compromise task execution; use of mobility aids; or evident functional limitations in daily activities. Additionally, women already engaged in structured physical training or taking medications likely to influence outcomes (e.g., hormone replacement therapy or osteoporosis treatments) were excluded.

The participants were divided into two groups: a control group and an exercise group. Randomization was performed using a simple random allocation sequence generated in RStudio software (version 2024.09.0+375) by an independent researcher who was not involved in recruitment or assessments. This ensured allocation concealment and minimized selection bias.

Participant recruitment followed a non-probabilistic approach. All participants were fully informed about the study’s objectives, potential risks, and expected discomforts, and participation was voluntary. Written informed consent was obtained from all participants prior to enrollment.

The sample consisted of 40 participants, with an average age of 68.68 ± 5.74 years and a body mass of 70.47 ± 14.88 kg, all of whom were women. The control group was composed of 20 participants, while the exercise group included 20 participants. All participants were biologically female. The study used “women” to reflect sex as reported by participants.

The volunteers were recruited from community projects and physical exercise programs promoted by the Polytechnic Institute of Bragança (IPB). Furthermore, baseline assessments included self-reported history of falls in the previous year and fear of falling, collected via a brief structured questionnaire. Few participants reported previous falls, and the majority did not report a significant fear of falling. Although these variables were not used as primary outcomes, they contributed to the initial characterization of fall risk in the study sample.

**Figure 1:**
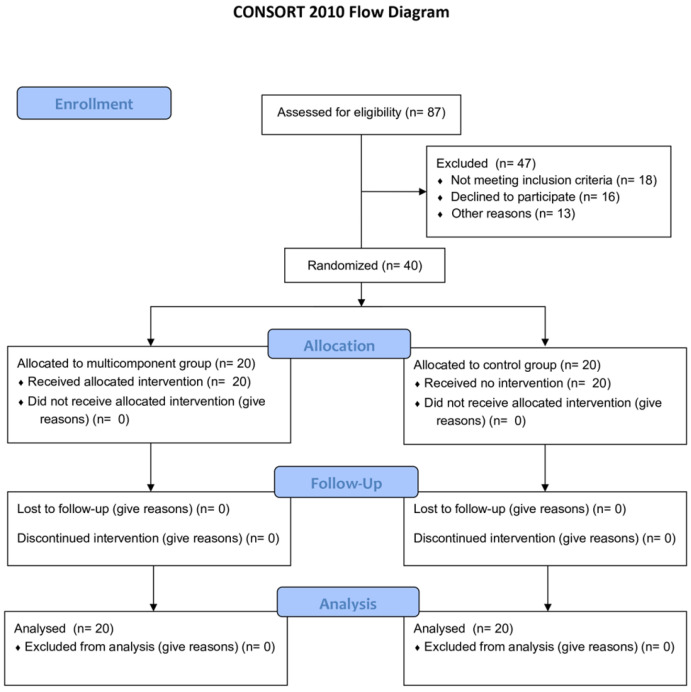
CONSORT diagram of the study.

Figure 1 describes the flow diagram of the study.

### 2.3. Procedures

#### 2.3.1. Anthropometry and Body Composition

Body weight was measured to the nearest 0.1 kg using a digital scale (SECA^®^; SECA GmbH & Co. KG, Hamburg, Germany), with participants dressed in light clothing and barefoot. Height was assessed using a stadiometer attached to the scale, measuring the vertical distance from the vertex of the head to the ground reference plane, with an accuracy of 0.1 cm. Body Mass Index (BMI) was calculated using the standard formula [weight (kg)/height^2^ (m^2^)] [60], and classified according to the World Health Organization (WHO) criteria (2010): normal weight (18.50–24.99 kg/m^2^), pre-obesity (25.00–29.99 kg/m^2^), obesity class I (30.00–34.99 kg/m^2^), and obesity class II (35.00–39.99 kg/m^2^) [61].

#### 2.3.2. Assessment of Functional Fitness

Functional fitness was assessed using the Rikli and Jones Senior Fitness Test [62], a validated test battery for older adults that evaluates key components such as muscular strength, flexibility, balance, agility, and aerobic capacity. The battery included the following six tests:

- Lower limb strength: 30 s chair stand test, measuring the number of full stands from a seated position.
- Upper limb strength: arm curl test using a 2 kg dumbbell, counting the number of repetitions performed in 30 s.
- Lower body flexibility: chair sit-and-reach test, with the distance (in centimeters) measured between the extended fingertips and the toes.
- Upper body flexibility: back scratch test, measuring the distance (in centimeters) between the middle fingers of each hand.
- Agility: Timed Up and Go (TUG) test, which records the time (in seconds) required to stand up from a chair, walk 3 m, turn around, return, and sit down.
- Aerobic endurance: 2 min step test, counting the number of steps completed while raising the knees to hip level.

This assessment protocol has been used in previous studies to evaluate the functional fitness of older adults [57,63].

#### 2.3.3. Multicomponent Training Program

**Figure 2:**
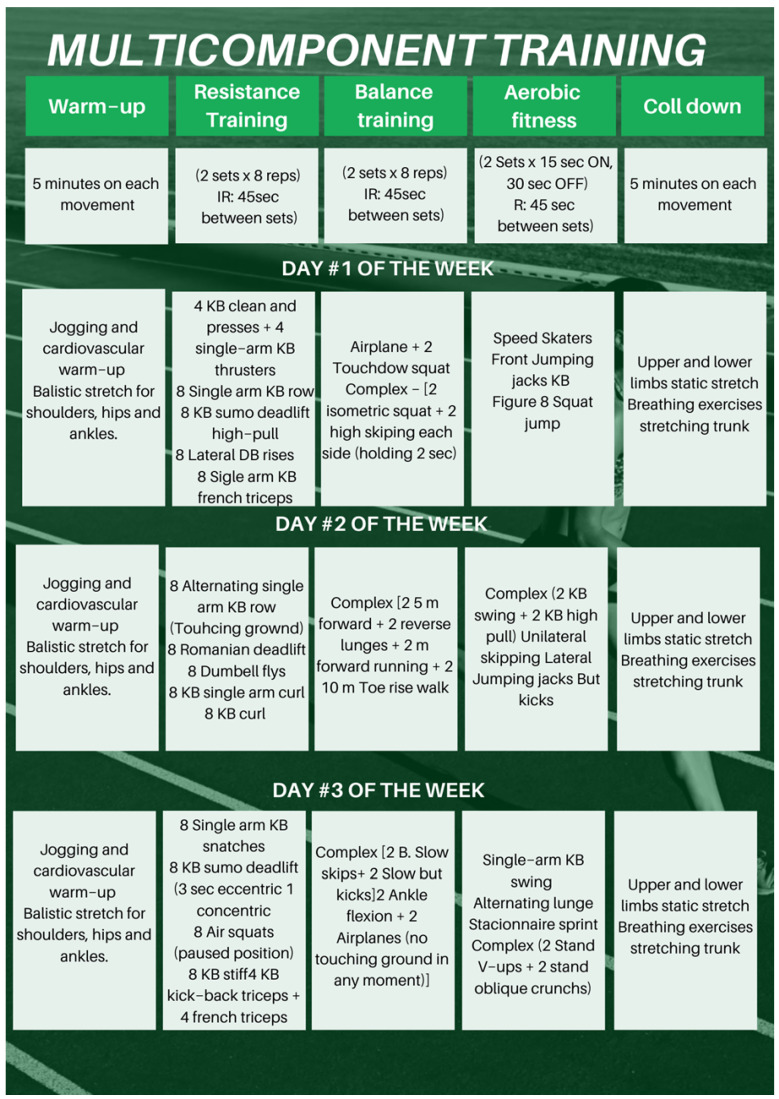
Multicomponent Training program.

The multicomponent training program was developed based on international guidelines for exercise in adult and older populations [64]. The intervention lasted 30 weeks and consisted of three 60 min sessions per week (Figure 2), delivered in a group setting. Each session followed a structured format:

- Warm-up (10 min): dynamic stretches, mobility exercises, and light jogging.
- Strength training (20 min): exercises using free weights, dumbbells, and kettlebells, targeting major muscle groups including upper limbs, lower limbs, and core.
- Balance and coordination training (10 min): activities such as single-leg exercises, plyometric movements, and tasks involving unstable surfaces.
- Aerobic training (15 min): dynamic and rhythmic movements like lateral jumps, jumping jacks, running in place, and heel raises.
- Cool-down (5 min): static stretches for upper and lower limbs and trunk, plus guided breathing exercises.

Sessions were supervised by certified physical education professionals and physiotherapists. Exercises were adapted to the individual functional capacity of each participant. Internal load was monitored weekly using the Borg Rating of Perceived Exertion (RPE) scale (6–20), with exercises adjusted to maintain perceived effort between 13 and 15 (moderate to intense).

Group sessions were held in a shared environment with more than 20 participants present, as the intervention coincided with other community-based multicomponent training projects conducted at the same facility. Despite the larger setting, each participant received individualized guidance and supervision from qualified physical education professionals and physiotherapists.

Participants assigned to the control group were instructed to maintain their usual lifestyle and not to engage in any new structured physical activity or exercise program during the 30-week study period. They received no intervention, physical guidance, or supervised sessions.

Monthly follow-up calls were made to ensure continued participation, confirm adherence to the non-intervention condition, and record any changes in physical activity, health status, or adverse events. These participants were included in the final analysis regardless of minor changes in behavior, provided they did not enroll in other exercise programs during the study period.

#### 2.3.4. Pre-Test and Post-Test Evaluations

The initial assessments (pre-test) were conducted before the start of the training program, and the final assessments (post-test) were carried out after the 30 weeks of intervention. The evaluations were conducted at the same times from 8:00 to 11:00 in the morning.

#### 2.3.5. Statistical Analyses

The collected data were analyzed using statistical methods, utilizing RStudio software (version 2024.09.0+375, Posit, PBC). Descriptive analyses, normality tests (Shapiro–Wilk and Levene’s), and comparisons between groups (ANOVA) were performed to evaluate the differences before and after the intervention [65,66,67]. The adopted significance level was *p* < 0.05 [68].

Initially, the Shapiro–Wilk test was applied to verify the normality of the data. For the variables that showed a normal distribution, a mixed ANOVA was used, which allows for the assessment of main effects (group and time) and the interaction between them (group × time), considering repeated measures within participants. This approach enables a robust analysis of the differences in body composition and functional fitness between the groups over time.

For the variables that did not follow a normal distribution, the non-parametric Brunner–Langer ANOVA was applied, suitable for data with non-normal distributions or variance heterogeneity, common characteristics in studies with specific populations, such as in this study. The analysis was conducted using the nparLD package in R software, generating Wald-type statistics (WTS) and ANOVA-type statistics (ATS), which allow for testing the absence of main effects and interactions without assuming parametric distributions.

## 3. Results

Table 1 shows the main characteristics and composition of the sample between the experimental group and the control group.

The Brunner–Langer nonparametric analysis for BMI showed that there was no significant group × time interaction (ATS = 1.370, *p* = 0.241), indicating that the variations over time did not differ significantly between the intervention and control groups. Additionally, no significant main effects were observed for group (ATS = 0.125, *p* = 0.723) or time (ATS = 1.458, *p* = 0.227). The effect size (Cohen’s d = 0.06) suggested a negligible and clinically irrelevant effect.

Descriptively, the intervention group exhibited a slight reduction in BMI, from 31.24 ± 5.96 kg/m^2^ (95% CI: 28.63 to 33.29) at baseline to 30.52 ± 6.13 kg/m^2^ (95% CI: 27.84 to 33.21) after the intervention, resulting in a Δ of –0.72 kg/m^2^. Conversely, the control group showed a small increase, from 29.67 ± 5.98 kg/m^2^ (95% CI: 27.04 to 32.29) to 29.83 ± 5.73 kg/m^2^ (95% CI: 27.32 to 32.34), corresponding to a Δ of +0.16 kg/m^2^. These findings indicate that the MTC program did not result in statistically or clinically meaningful changes in BMI among the participants.

The mixed ANOVA for upper limb flexibility, assessed using the Back Scratch Test, revealed a significant group × time interaction (F(1,38) = 19.35, *p* < 0.001, η^2^_p_ = 0.951), indicating that the groups responded differently over time. No significant main effects were observed for group (F(1,38) = 0.35, *p* = 0.556, η^2^_p_ = 0.261) or time (F(1,38) = 2.37, *p* = 0.132, η^2^_p_ = 0.703), suggesting that the observed differences were primarily due to the interaction effect.

Descriptively, the intervention group improved from −11.68 ± 8.40 cm (95% CI: −15.45 to −7.90) at baseline to −7.95 ± 9.14 cm (95% CI: −12.06 to −3.83) post-intervention, resulting in a Δ of +3.73 cm. In contrast, the control group showed a slight decline, from −7.38 ± 10.03 cm (95% CI: −11.88 to −2.87) to −8.60 ± 10.73 cm (95% CI: −13.43 to −3.77), with a Δ of −1.22 cm. These findings suggest a positive effect of the MTC program on upper limb flexibility in postmenopausal women.

The mixed ANOVA for upper limb strength, assessed using the Arm Curl Test, revealed a significant group × time interaction (F(1,38) = 42.59, *p* < 0.001, η^2^_p_ = 0.529), indicating that the effects of the intervention differed between the groups over time. Additionally, a significant main effect of time was observed (F(1,38) = 16.47, *p* < 0.001, η^2^_p_ = 0.302), suggesting an overall improvement in upper limb strength from pre- to post-intervention. However, the main effect of group was not significant (F(1,38) = 0.63, *p* = 0.431, η^2^_p_ = 0.016), indicating that the groups did not differ significantly when analyzed independently of time.

Descriptive results showed that the intervention group improved from 24.60 ± 8.85 repetitions (95% CI: 20.62 to 28.58) at baseline to 29.95 ± 8.78 repetitions (95% CI: 26.00 to 33.90) post-intervention, resulting in a Δ of +5.35 repetitions. In contrast, the control group declined from 31.65 ± 9.94 repetitions (95% CI: 27.18 to 36.12) to 27.45 ± 8.77 repetitions (95% CI: 23.51 to 31.39), corresponding to a Δ of −4.20 repetitions. These findings reinforce the effectiveness of the multicomponent training program in enhancing upper limb strength in postmenopausal women.

The mixed ANOVA for lower limb strength, assessed using the Chair Stand Test, revealed a significant group × time interaction (F(1,38) = 75.38, *p* < 0.001, η^2^_p_ = 0.987), indicating that the changes in performance over time were different between the intervention and control groups. A significant main effect of time was also found (F(1,38) = 21.72, *p* < 0.001, η^2^_p_ = 0.956), suggesting an overall improvement in lower limb strength across time points. However, the main effect of group was not significant (F(1,38) = 0.06, *p* = 0.811, η^2^_p_ = 0.054), showing that there was no significant difference between groups when time was not considered.

Descriptively, the intervention group improved from 19.75 ± 4.24 repetitions (95% CI: 17.84 to 21.66) at baseline to 24.00 ± 4.31 repetitions (95% CI: 22.06 to 25.94) post-intervention, with a Δ of +4.25 repetitions. In contrast, the control group declined from 22.75 ± 6.60 repetitions (95% CI: 19.78 to 25.72) to 20.15 ± 6.70 repetitions (95% CI: 17.14 to 23.16), corresponding to a Δ of −2.60 repetitions. These results support the effectiveness of the MTC program in promoting significant gains in lower limb strength among postmenopausal women.

The Brunner–Langer nonparametric analysis for aerobic endurance, assessed using the 2 min Step Test, revealed a significant group × time interaction (ATS = 26.42, *p* < 0.001), indicating that the response to the intervention differed between the groups. A significant main effect of time was also observed (ATS = 5.50, *p* = 0.019), suggesting changes in aerobic capacity regardless of group assignment. However, the main effect of group was not statistically significant (ATS = 0.605, *p* = 0.436). The effect size was moderate (Cohen’s d = 0.426), indicating a meaningful impact of the intervention on aerobic endurance.

Descriptively, the intervention group increased its average step count from 114.50 ± 52.21 (95% CI: 91.02 to 137.98) at baseline to 136.60 ± 51.60 (95% CI: 113.40 to 159.80) after the intervention, resulting in a Δ of +22.10 steps. In contrast, the control group showed a decrease from 139.90 ± 40.00 (95% CI: 121.91 to 157.89) to 130.30 ± 35.22 (95% CI: 114.47 to 146.13), with a Δ of −9.60 steps. These results suggest that the multicomponent training program had a positive effect on aerobic endurance in postmenopausal women.

The Brunner–Langer nonparametric analysis for lower limb flexibility, assessed using the Sit-and-Reach Test, revealed a significant group × time interaction (ATS = 34.232, *p* < 0.001), indicating that the effects of the intervention differed significantly between the groups over time. A significant main effect of time was also found (ATS = 5.078, *p* = 0.024), suggesting improvements in flexibility regardless of group. However, the main effect of group was not significant (ATS = 0.020, *p* = 0.885). The effect size was moderate (Cohen’s d = 0.437), indicating a meaningful intervention impact on this variable.

Descriptive analysis showed that the intervention group improved from 4.85 ± 6.75 cm (95% CI: 1.82 to 7.88) at baseline to 7.82 ± 6.85 cm (95% CI: 4.74 to 10.90) after the intervention, resulting in a Δ of +2.97 cm. In contrast, the control group declined from 6.78 ± 7.29 cm (95% CI: 3.49 to 10.06) to 5.10 ± 7.80 cm (95% CI: 1.59 to 8.61), with a Δ of −1.68 cm. These findings suggest that the MTC program contributed to meaningful improvements in lower limb flexibility in postmenopausal women.

The Brunner–Langer nonparametric analysis for agility, assessed using the Timed Up and Go (TUG) test, revealed a significant group × time interaction (ATS = 6.792, *p* = 0.009), suggesting that the changes in agility over time differed significantly between the intervention and control groups. No significant main effects were observed for group (ATS = 0.768, *p* = 0.380) or time (ATS = 0.421, *p* = 0.516). The effect size, expressed as Cohen’s d, was −0.388, indicating a small-to-moderate effect in favor of the intervention group (noting that lower times represent better performance).

Descriptively, the intervention group improved by reducing its average TUG time from 4.87 ± 0.88 s (95% CI: 4.47 to 5.27) at baseline to 4.60 ± 0.44 s (95% CI: 4.40 to 4.80) post-intervention (Δ = −0.27 s). Conversely, the control group showed a decline in performance, increasing from 4.74 ± 0.73 s (95% CI: 4.41 to 5.07) to 4.99 ± 0.78 s (95% CI: 4.63 to 5.34), with a Δ of +0.25 s. These results highlight the effectiveness of the MTC program in enhancing agility among older women.

**Figure 3:**
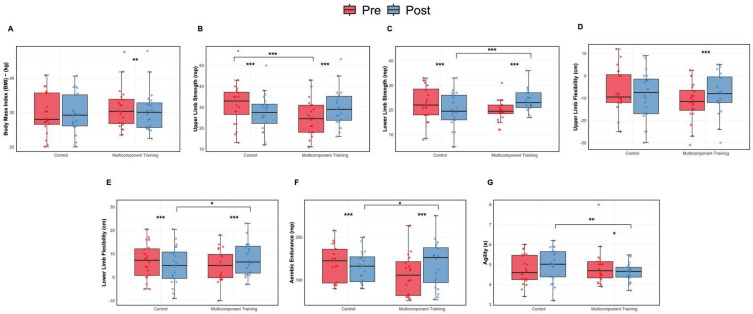
Body composition and functional fitness assessment: pre and post intervention comparisons. (**A**) Body Mass Index (BMI). (**B**) Upper limb strength. (**C**) Lower limb strength. (**D**) Upper limb flexibility. (**E**) Lower limb flexibility. (**F**) Aerobic endurance. (**G**) Agility. Data are presented as boxplots showing median and individual values. * *p* < 0.05, ** *p* < 0.01; *** *p* < 0.001.

These results highlight the effectiveness of the MTC program in enhancing agility among older women (Figure 3).

## 4. Discussion

The objective of this study was to evaluate the role of multicomponent exercise in fall prevention and the improvement of functional fitness in older women, with a particular focus on the Portuguese population. One of the main functional outcomes assessed was mobility, evaluated through the Timed Up and Go (TUG) test. This test is a widely used and validated tool to assess functional mobility and dynamic balance in community-dwelling older adults [69]. Although it is commonly included in fall risk assessments, its ability to predict falls is limited in individuals with preserved mobility [70].

However, clinical cut-off points have been proposed in the literature. For instance, individuals who take longer than 13.5 s to complete the TUG test are considered at increased risk of falls, with both sensitivity and specificity reported at 87% [71]. Therefore, the improvements observed in TUG performance in this study should be interpreted primarily as gains in functional mobility and balance, rather than conclusive evidence of reduced fall risk.

The results demonstrated that participants who engaged in the multicomponent training program showed a significant improvement in TUG performance, indicating gains in agility and motor control. These findings suggests that the exercise program was effective in enhancing the functional capacity of the participants, making them more capable of performing daily activities with a lower risk of falls. Previous studies have already demonstrated that interventions based on physical exercises, especially those that include strength, balance, and coordination training, are essential in fall prevention among older adults [49,50,56,57].

In addition to the TUG, the results also indicated improvements in lower limb strength, flexibility, and aerobic endurance. The significant interaction between group and time for these outcomes reinforces the idea that a well-structured exercise program can not only maintain but also reverse age-related functional decline [72]. The improvement in lower limb strength is particularly relevant, as muscle weakness is directly associated with the inability to respond appropriately to postural disturbances, increasing the risk of falls [73]. These results are consistent with previous findings that highlight the role of strength gains in enhancing postural stability and reducing fall risk in older adults [74,75,76].

The literature indicates that multicomponent training programs are more effective in fall prevention [77,78], when compared to interventions focused on just one component, such as aerobic or strength training in isolation [29,79]. In the meta-analysis conducted by Sadaqa et al., the combination of strength, balance, and agility exercises proved to be an effective strategy to improve functionality and reduce risk factors for falls, reinforcing the need to promote this type of intervention in aging populations [29].

It is worth noting that several functional fitness tests used in this study have established cut-off values that are associated with an increased risk of falling. For instance, in the Timed Up and Go (TUG) test, values above 13.5 s have been linked to a higher fall risk in older adults [71]. Similarly, reduced performance in lower limb strength and flexibility tests has been associated with impaired balance and mobility [29]. Therefore, the significant improvements observed in TUG performance and other functional parameters in the intervention group may reflect not only statistical but also clinically meaningful reductions in fall risk.

Despite the relevant findings, this study presents some limitations. The sample size, while sufficient to detect differences, limits generalizability. Moreover, the exclusive inclusion of women prevents assessment of possible sex-related differences in training response, which should be explored in future research. External factors such as physical activity outside the program, diet, and session adherence were not fully controlled and may have influenced the outcomes.

In interpreting these findings, several methodological limitations should also be considered. Although the study followed a single-blind design with blinded outcome assessors, participants and instructors were aware of group allocation, which may have introduced performance or expectancy bias. Additionally, the control group did not receive a placebo or structured intervention, and incidental physical activity was not systematically monitored. While exercise intensity was guided by the Borg Rating of Perceived Exertion and adjusted individually, objective measures of intensity (e.g., heart rate, workload) were not recorded. The sample consisted solely of community-dwelling women, further limiting generalizability. Finally, although improvements were seen in functional outcomes associated with fall risk, the study did not directly assess fall incidence. These factors must be taken into account when interpreting the results and drawing conclusions about the broader applicability of the intervention.

Future studies could include larger, more diverse samples and adopt a longitudinal design to assess the long-term effects of multicomponent training. Exploring its application in different settings—such as community centers, clinics, or institutions—and among populations with reduced mobility or prior falls may enhance the understanding of its effectiveness. The integration of technologies, like motion sensors or mobile apps, may also support monitoring and adherence.

Finally, combining multicomponent training with other interventions, such as nutritional or cognitive strategies, may further enhance its benefits. This multifaceted approach can be key to promoting healthier, more independent aging and contributing to the sustainability of healthcare systems.

## 5. Conclusions

This study demonstrated that multicomponent exercise is an effective strategy to improve functional fitness in older women. The observed gains in mobility, strength, balance, and endurance—particularly the improvement in TUG performance—highlight the positive impact of this approach on physical function. These findings support the use of structured multicomponent training as a practical and beneficial intervention to enhance functional capacity and promote autonomy during aging. While improvements were seen in parameters associated with fall risk, further research is needed to determine whether such interventions lead to a direct reduction in fall incidence.

## Figures and Tables

**Table 1:** Descriptive statistics for group composition, age, and BMI.

Group (n)	Age (Mean ± SD)	Median	Min./Max. Age	BMI (Mean ± SD)	Median	Min./Max. BMI	*p*-Value
Control (20)	68.5 ± 5.24	67	61/82	31.25 kg/m^2^ ± 5.97	30.34	23.47/47.58	0.89
Multicomponent Training (20)	68.8 ± 6.35	68.5	60/86	29.67 kg/m^2^ ± 5.98	28.01	20.10/40.78	0.40

SD (Standard Deviation); Min (Minimum); Max (Maximum).

## Data Availability

The data supporting the findings of this study are not publicly available due to privacy or ethical restrictions but can be provided by the corresponding author upon reasonable request.
